# Collaboration Between Public Health and Law Enforcement: New Paradigms and Partnerships for Bioterrorism Planning and Response

**DOI:** 10.3201/eid0810.020400

**Published:** 2002-10

**Authors:** Jay C. Butler, Mitchell L. Cohen, Cindy R. Friedman, Robert M. Scripp, Craig G. Watz

**Affiliations:** * Centers for Disease Control and Prevention, Atlanta, Georgia, USA; † Federal Bureau of Investigation, Washington, D.C., USA

**Keywords:** police power, anthrax, quarantine, bioterrorism response, *Bacillus anthracis*, law, criminal investigation, Federal Bureau of Investigation

## Abstract

The biological attacks with powders containing *Bacillus anthracis* sent through the mail during September and October 2001 led to unprecedented public health and law enforcement investigations, which involved thousands of investigators from federal, state, and local agencies. Following recognition of the first cases of anthrax in Florida in early October 2001, investigators from Centers for Disease Control and Prevention (CDC) and the Federal Bureau of Investigation (FBI) were mobilized to assist investigators from state and local public health and law enforcement agencies. Although public health and criminal investigations have been conducted in concert in the past, the response to the anthrax attacks required close collaboration because of the immediate and ongoing threat to public safety. We describe the collaborations between CDC and FBI during the investigation of the 2001 anthrax attacks and highlight the challenges and successes of public health and law enforcement collaborations in general.

 Public health and law enforcement agencies become involved in the investigation of a possible bioterrorism event under different circumstances. Such events fall into one of two categories: overt and covert. In the overt event, the perpetrator announces responsibility for something (for example, release of an agent) or the nature of the event reveals itself (i.e., the 1995 sarin attack by the Aum Shinrikyo in the Tokyo subway). In the overt attack, usually law enforcement first detects the event, leads the initial response, and notifies public health officials (Figure 1). If persons are ill or preventive health services are indicated, public health will also become involved in the emergency response.

**Figure 1:**
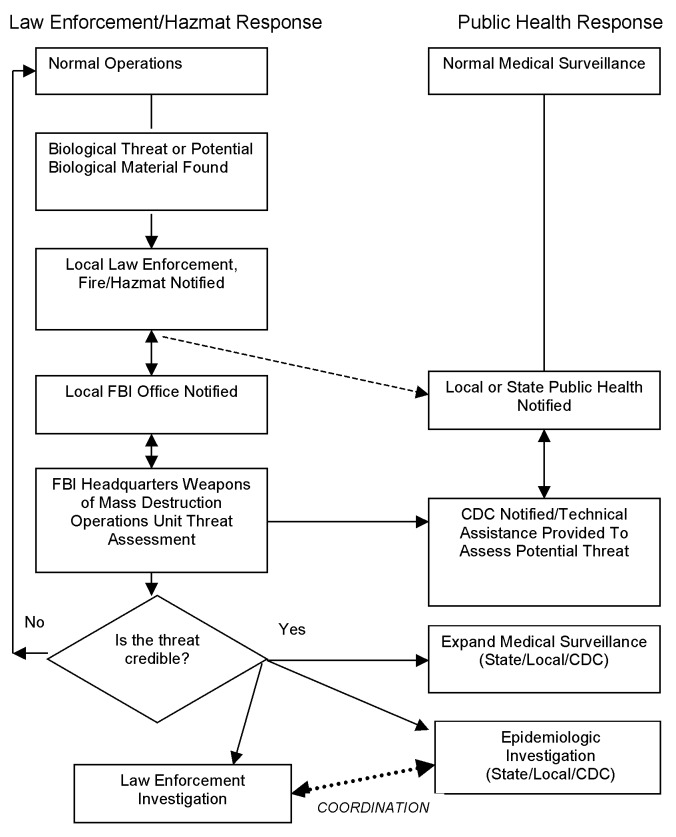
Flow chart of response to overt bioterrorism.

In contrast, the covert event is characterized by an unannounced or unrecognized release in which the presence of ill persons may be the first sign of an attack. In the covert attack, criminal intent may not be apparent until some time after illnesses are recognized. This distinction is important for establishing and understanding the partnership between public health and law enforcement. The overt event is clearly a crime, and the site of the incident is a crime scene. As a result, access to the area may be restricted so that evidence can be collected pursuant to the criminal investigation. Under federal statute (Title 18, U.S.C. Section 2332[a]), any threatened use of a disease-causing organism directed at humans, animals, or plants is a crime, regardless of whether the perpetrator actually possesses a disease-causing agent. In addition, as a result of a change in the Bioterrorism Weapons Anti-Terrorism Act contained in the USA PATRIOT Act of 2001 and codified in Title 18 USC Section 175(b), knowingly possessing a biological agent, toxin, or delivery system which cannot be “justified by a prophylactic, protective, bona fide research, or other peaceful purpose” can result in arrest, prosecution, and fines and/or imprisonment for up to 10 years. This new provision shifts the burden of proof onto the person or persons who are in possession of dangerous biological agents to prove they have the material for legitimate purposes.

 The covert event may not be initially recognized as an attack, and public health generally first recognizes the problem and leads the initial inquiry (Figure 2). The early response will focus on diagnosis, medical care, and epidemiologic investigation. The intentional and criminal nature of the event may not be immediately evident, and notification of law enforcement may be delayed as a result. A 1985 outbreak of gastroenteritis in Oregon that was caused by a religious cult contaminating multiple salad bars with salmonella was initially thought to be a natural event (1). The crime was only recognized after the cult’s leader accused other cult members of the attack and publicly called for an investigation. The subsequent criminal investigation confirmed the role of cult members in the outbreak.

**Figure 2:**
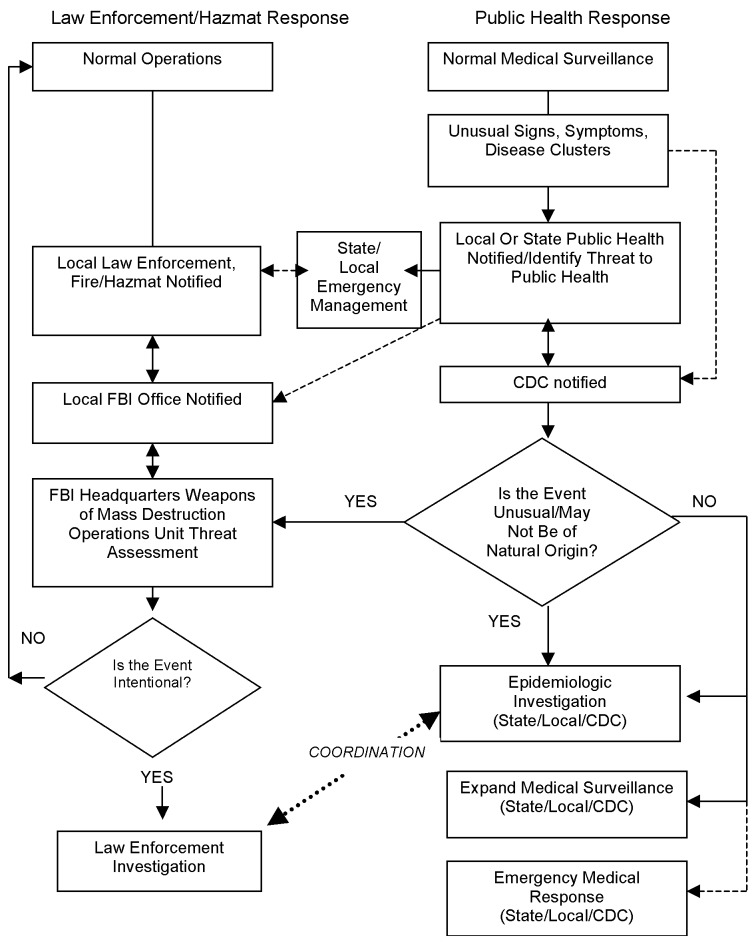
Flow chart of response to covert bioterrorism.

Microbiologic factors may also provide the first clue of the criminal intent of a disease outbreak. In 1996, an outbreak of gastroenteritis among staff in the laboratory of a large medical center was caused by *Shigella dysenteriae* type 2, a pathogen that is unusual in the United States (2). An epidemiologic investigation linked infection with eating pastries that had been placed in the laboratory break room. *S. dysenteriae* type 2 matching the laboratory’s stock strain by pulsed-field gel electrophoresis was recovered from ill laboratory workers and from an uneaten pastry. A portion of the laboratory’s stock strains was missing, and subsequent criminal investigation identified a disgruntled former laboratory employee as the perpetrator.

 The anthrax attacks in September and October 2001 provide examples of both overt and covert events and highlight the different ways that public health and law enforcement agencies become involved in investigating bioterrorist attacks. The first case that was recognized in Florida in early October could have represented a natural event and was initially investigated as a public health issue (3,4). However, law enforcement officials were notified and involved in the initial investigation because of the rarity of inhalational anthrax in the United States (5,6), because *B. anthracis* has known potential as a biological weapon (7,8), and because of increased vigilance for a possible bioterrorist attack after the events of September 11. Once the intentional nature of the event was made evident by the second suspected case of inhalational anthrax in Florida, law enforcement involvement increased dramatically. The receipt of an envelope containing a threatening letter and *B. anthracis* at the Hart Senate Office Building on October 15, 2001, required that the site be handled as a crime scene, and the role of public health was primarily consequence management and technical assistance to the Federal Bureau of Investigation (FBI) and other law enforcement officials.

## Similarities and Differences

 Although both public health and law enforcement protect the public, the approach and nature of the work performed in the two disciplines are quite different. The similarities and differences in public health and law enforcement investigations have to be understood and coordinated so that both can be most effective (Table). Public health investigations generally take an inductive approach. Persons are interviewed, data are collected, hypotheses are developed to explain transmission, and epidemiologic and laboratory studies are conducted to test these hypotheses. If the studies confirm the hypothesis, prevention and control strategies are developed, implemented, and evaluated. All this work is held to the standard of scientific peer review, generally through presentation of data at scientific meetings and publication in a scientific journal.

**Table:** Differences in public health and law enforcement investigations

Characteristics	Public health	Law enforcement
Method of event recognition	Event detected through public health surveillance or calls from clinicians	Event announced by attacker or is evident
Challenges to event recognition	Few clinical syndromes that are clearly the result of bioterrorist attack; difficulty distinguishing between disease of natural origin and bioterrorism attack	Large number of hoaxes and noncredible threats not associated with an actual bioterrorist attack; delay in notification of possible event by public health; “copycat” threats or attacks (9)
Initial data collection	Hypothesis generation, “shoe-leather epidemiology”	Questioning of witnesses and suspects, follow-up of tips and intelligence information
Confirmatory data collection and analysis	Controlled epidemiologic studies	Collection and organization of evidence
Data validation	Presentation for scientific peer review	Indictment, arrest, and conviction
Goal of investigation	Effective disease prevention and control measures	Prevention and deterrence of future attacks

On the other hand, the law enforcement investigation takes a deductive approach and is held to a very different standard. Witnesses and potential suspects are interviewed, leads are developed and pursued, and all available evidence is collected, identified, and tracked. If evidence is adequate, the suspected perpetrator is identified, arrested, and prosecuted. The work of law enforcement is held to legal standards. Thus, while the public health investigator’s aim is to collect data that will withstand the scrutiny of subject matter experts and the global scientific community, with the ultimate goal of developing effective control measures, the law enforcement investigator’s goal is gathering evidence that will meet constitutional standards and withstand legal challenges to obtain a conviction.

 The differing nature of the work and standards to which the work is held can pose difficulties on occasion when public health and law enforcement officials conduct joint investigations. In high-profile investigations, such as the anthrax attacks in 2001, these differences can be exaggerated by public perceptions and media portrayals of public health and law-enforcement investigative methods. The issues become even more complex when events involve multiple geographic areas or organizations that have overlapping responsibilities. These difficulties can be addressed within the public health and law enforcement communities by understanding each other’s approaches, by communicating effectively, and by making thoughtful preparations, including testing the system through exercises (10–12). These measures will improve collaboration during crises. The adage that “an emergency is a bad time to begin exchanging business cards” applies. During the investigations of the anthrax attacks in 2001, preexisting relationships between FBI field offices and state and local public health officials improved communications for field investigations and facilitated the public health response (M. Layton, New York City Health Department, pers. comm.).

 Preexisting relationships were particularly important for coordinating microbiologic testing of environmental and clinical samples, which were critical to both investigations. Before the 2001 anthrax incidents, the Centers for Disease Control and Prevention (CDC) and FBI began working together to develop notification procedures for possible bioterrorism events and to establish the Laboratory Response Network (LRN) for Bioterrorism, a multilevel network connecting local and state public health laboratories with advanced capacity public health and military laboratories (13). The federal, state, and local collaborative effort of law enforcement and public health that developed the LRN is the result of predicting the need for validated tests that would be consistent with evidentiary requirements. A uniform set of laboratory protocols, based on established procedures and reagents, facilitates the introduction of test results into a court of law, thereby limiting evidentiary challenges that may result from the use of different testing methods or analyses. Because clinical specimens are referred to LRN laboratories for analysis, the LRN also serves as a front-line resource and detection mechanism for identifying a potential covert attack. The 2001 anthrax incidents demonstrated the importance of the LRN in responding to a biological attack and revealed the need to expand its laboratory capacities.

## New Partnerships, New Paradigms

 Although federal, state, and local public health plans for responding to bioterrorism contributed to a state of readiness that would not have been possible only a few years earlier, the response to the 2001 anthrax attacks required venturing into unfamiliar territory for many public health and law enforcement officials. Historically, most terrorist attacks on Americans have involved use of explosives (14), and investigations have been conducted by FBI and other law enforcement agencies, while public health involvement has generally been limited to ensuring safe working conditions for investigators and aid workers and assessment of the acute and long-term physical and mental health effects (15–19).

 For many public health officials, responding to the rising threat of bioterrorism and recent attacks has necessitated a steep learning curve. Public health investigators usually approach infectious disease outbreaks as naturally occurring events, rather than the result of criminal acts, and they are unaccustomed to working closely with law enforcement personnel (11,12). Additionally, national security clearance has not been a requirement for most public health professionals, for whom the clearance process is unfamiliar. During 2001, few public health investigators had equipment such as secure telephone and fax lines necessary for sharing sensitive information with law enforcement officials. Confidentiality is maintained in public health investigations for the purpose of protecting sensitive patient medical information rather than national security. In law enforcement, confidentiality is also maintained to protect informants and witnesses and to preserve the integrity of the case for prosecution. Before 2001, most public health officials were not familiar with the principles of maintaining the chain of custody of specimens submitted for microbiologic testing so that laboratory results could be used for criminal prosecution.

Collaboration with law enforcement officials generally has not been recognized as beneficial or desirable in public health. The presence of law enforcement officers has been thought to compromise the collection of sensitive medical information (e.g., illegal drug use). Indeed, some degree of separation from law enforcement may be advantageous for obtaining complete and accurate data during public health investigations. Public health services are vitally needed by medically underserved communities, where suspicion of law enforcement agencies is intense, and collaboration with law enforcement agencies has even been described as “destructive to public health efforts” (20). However, the role of law enforcement in investigating potential bioterrorism incidents requires interviewing all potential witnesses and victims. Separate questioning by law enforcement and public health investigators may lead to conflicting statements by the interviewee, jeopardizing the admissibility of those statements in subsequent judicial proceedings. A process should be established whereby joint interviews by public health and law enforcement officials are conducted, with opportunity for confidential communications with public health officials regarding specific health-related issues that the interviewee may be unwilling to share with law enforcement personnel present. Both law enforcement and public health must recognize that the sharing of information can be crucial for identifying persons who have been exposed to dangerous agents and may be in need of prevention services such as chemoprophylaxis or vaccination.

 Law enforcement is now increasingly focused on prevention of terrorist acts, requiring a new partnership with the public health and medical community. The steps necessary to identify a potential covert bioterrorism attack include a close coordination between those who collect and analyze medical and syndromic surveillance information with the law-enforcement community’s intelligence and case-related information. The best method for timely detection of a covert bioterrorist attack is early communication between the two communities and recognition of the extent and origin of the threat. For the FBI, this recognition requires conducting a threat/credibility assessment, a process coordinated by the Weapons of Mass Destruction Operations Unit, FBI Headquarters, in conjunction with CDC and other federal agency experts. The FBI threat assessment is necessary to determine whether the circumstances may be the result of an intentional or criminal act, warranting law enforcement involvement. In some cases, a joint FBI–public health investigation is necessary to gather facts to determine whether a criminal act has actually occurred.

 The work of CDC and FBI during the ongoing anthrax investigation highlights the opportunity for collaboration between public health and law enforcement. During several of the anthrax field investigations in 2001, investigators from FBI or local law enforcement were paired with an epidemiologist during interviews of possible case-patients and exposed persons, which allowed a multidisciplinary approach to collecting, processing, and sharing pertinent information. Because of different training backgrounds and professional experiences, law enforcement and public health interviewers may recognize and note different information or clues that could aid in identifying the source of the infection and its perpetrator(s). Additionally, the concurrent interviews reduced the number of times persons had to be questioned. Since October 12, a senior medical epidemiologist from the National Center for Infectious Diseases, CDC, has been assigned to FBI headquarters or to the Washington field office to help facilitate communication of information between the agencies and to provide on-site medical and public health consultation as threats of new possible biological attacks are assessed.

## Conclusion

 Partnership between public health and law enforcement is prerequisite to sound bioterrorism planning and response. Each group can add value to the work of the other. At the federal level, both CDC and FBI have unique perspectives and expertise that can benefit the other. For the FBI, CDC offers medical and laboratory consultation and collaboration combined with national and international public health connections. For CDC, the FBI offers criminology expertise, forensic laboratory collaboration, and access to intelligence information, along with national and international law enforcement connections. Each agency offers a unique perspective and opportunities to share information. Similar partnerships exist or should exist at the state and local level. Public health and law enforcement must understand each other’s work, standards, and culture. The heat of an investigation can strain even the best relationships. Thus, public health and law enforcement need to increase mutual collaboration and understanding before they are thrown together in the response to a biological attack. To this end, liaison personnel are needed who have some degree of cross-training in the public health aspects of communicable diseases and in law enforcement and criminal investigations.
